# Interrelationship between subjective wellbeing and health

**DOI:** 10.1186/s12889-024-19676-3

**Published:** 2024-08-14

**Authors:** Qaqambile Mathentamo, Nozuko Lawana, Besuthu Hlafa

**Affiliations:** 1 Department of Economics, Faculty of Management and Commerce, University of Fort Hare, East London, South Africa; 2 University of the Witwatersrand, Johannesburg, South Africa; 3 Department of Economics, Human Sciences Research Council, Pretoria, South Africa; 4 Department of Economics, Economics & Finance, Tshwane University of Technology, Tshwane, South Africa

**Keywords:** Subjective well-being, Happiness, Life satisfaction, Health, Determinants

## Abstract

**Background:**

Subjective wellbeing (SWB) and health are important facets of any person’s life, and they tend to influence each other. This importance is reflected in the vastness of literature aiming to explore this association. However, most of this literature is based on sampling national population which may present different population characteristics to those of a province. Thus, the paper aims to investigate if the association between perceived health and SWB is moderated by population characteristics at a provincial level. We intend to add value to subjective wellbeing and health literature by reviewing the relationship between SWB and health in the Eastern Cape stratified by age, gender, rural and urban and different income classifications.

**Methods:**

Different population characteristics tend to associate to subjective wellbeing and health differently, therefore influencing how these two variables influence each other. Thus, the paper aims to investigate if the association between perceived health and SWB is moderated by population characteristics at a provincial level. Utilising the South African National Income Dynamics Survey from 2008 to 2017, this study examined the relationship between subjective wellbeing and health using a random effects model stratified according to aforementioned population characteristics.

**Results:**

A better perceived health status is associated with higher subjective wellbeing. A better subjective wellbeing is also associated with a higher health status. Determinants of subjective wellbeing and health associate with these variables differently besides income and employment which led to higher subjective wellbeing and health. Health associates to subjective wellbeing different across populations characteristics.

**Conclusion:**

Subjective wellbeing and health are interdependent as reflected in the World Health Organisation (WHO) and United Nation (UN) statements. Higher income and level of education and being employed is associated with both higher SWB and health. Therefore, improving these economic outcomes maybe associated with an improvement in well-being and health as desired by WHO and UN. Provinces differ, and different population characteristics tend to associate with subjective wellbeing and health differently, therefore influencing how these two variables influence each other. Health improvement policies must consider subjective wellbeing. Well-being improvement policies need to be cognisant of the differences in provincial and population characteristics.

## Background

Individuals prioritise and classify “subjective well-being” (SWB) and “health” as one of the most important life’s purposes. This is supported by the assertion which states that time memorial philosophers regarded happiness as the decisive impetus for human engagement [1–3]. Yet, one cannot realise SWB without being healthy vice versa. This interdependence is clearly stated in the World Health Organisation’s (WHO) constitution cited below:

**Table:** 

“Health is a state of complete physical, mental, and social well-being and not merely the absence of disease or infirmity” https://www.who.int/about/governance/constitution.	

WHO’s statement do not just inform us about the importance of health but the dependence of health on well-being. There is a clear link between (social) SWB and health where realisation of health is realisation of SWB. This is also reflected in the United Nation’s (UN) Sustainable Development Goal number three (SDG 3) which aims for the realisation of healthy lives and promotion of well-being for all. Besides their interdependence, SWB and health also have the potential of influencing productivity, wages or earnings and employment [4, 5].

Hence, there is a bidirectional relationship between health and SWB [3, 6–9] making it hard to infer causality [10, 11]. Different population characteristics tend to be associated with SWB and health differently, therefore influencing how these two variables influence each other. Thus, we aim to investigate if the association between perceived health and SWB is moderated by population characteristics at a provincial level. We hypothesise that perceived health’s association to SWB may differ per population characteristics at a provincial level. The paper will add value to this body of literature by reviewing the relationship between SWB and Health in the Eastern Cape stratified by age, gender, rural and urban [10, 11], and different income levels.

The paper’s investigation of the relationship between these variables in a South African context is vital. This research has the potential of being beneficial to policymakers and South African citizens. Policymakers can benefit from this research as Gross Domestic Product (Gross National Product) is increasingly considered “an inadequate measure of national progress” [12]. Policies that aim to improve health ought to be cognisant of people’s subjective wellbeing. The citizens at large stand to gain from this study’s findings since most studies published under subjective well-being usually “attract considerable interest, especially among the general public” [12]. The paper can also inform economic outcomes related to labour markets, economic growth, and development.

The paper is outlined as follows: section one reviews the literature on SWB and health. Section two outlines the methodology used to study the interrelationship between SWB and health. Section three and four provides detailed results and discussion, respectively. The final section presents the conclusion.

### Literature review 

Subjective well-being and health are two distinct economic concepts, which are influenced by determinants separately, however, one cannot be fully realised without the other. To realise subjective well-being, one must be healthy as reflected in the WHO’s statement and UN’s SG3. Thus, we looked at how literature scrutinise these concepts separately. We also briefly outlined how SWB and health influence each other and we ended off with our contribution.

Subjective well-being anchors around two philosophical foundations – eudaimonia and hedonism. Eudaimonia means realisation of one’s true nature or potential, whereas hedonism is the seeking of pleasure and avoiding of pain [3, 13, 14]. Hedonism informs most research on economic of happiness since literature tend to study which socio-economic elements increase or decrease subjective well-being.

Most researchers use subjective well-being as an “umbrella term” [2] and measure it using happiness or life satisfaction questions. This is guided by the premise that subjective well-being under hedonic psychology has three components: life satisfaction, positive effects, and avoidance of negative effects [3, 8, 9, 12, 13]. Plainly put, most researchers measure subjective well-being using life satisfaction or happiness questions which differ in structure and scale [15].

Example of the questions:

**Table:** 

World Values Survey: “All things considered, how satisfied are you with your life as a whole these days?” The General Social Survey (GSS): “Taken all together, how would you say things are these days? Would you say that you are very happy, pretty happy, or not too happy?” European Social Survey (ESS) “How happy would you say you are? [rate] happiness on a scale from 0 (‘extremely unhappy’) to 10 (‘extremely happy’)” [16]. “A typical question is, “Overall, how satisfied are you with your life these days?” People reply on a scale of 0-10 (0= completely dissatisfied, 10= completely satisfied)” [17].	

Subjective well-being, happiness and life satisfaction have different meanings when they are precisely communicated [1]. Happiness and life satisfaction may associate with different determinants of subjective well-being differently [18]. However, researchers use subjective well-being, happiness and  life satisfaction interchangeably since happiness and life satisfaction correlate closely with each other [6]. Thus, this paper measures subjective well-being using life satisfaction questions that are found within the dataset used in this paper. The paper also makes use of these terms interchangeably driven by the premise that subjective well-being is an umbrella term which encompasses happiness and life satisfaction.

SWB survey questions are mainly single item since it is costly to have multi-item questions [12]. Hence, a wide range of researchers use single item even though there is criticism: cannot quantify variance, less consistent overtime and do not cover all element of SWB [1, 8, 10, 11, 15, 19–22]. The paper will follow suite and use a single item question. Our database is based on single question since it is cost effective and convenient to use a single question. Most happiness or SWB questions are single item questions even the World Happiness report is based on a single item question.

The study of happiness has developed a lot since the Easterlin Paradox attributed to Richard Easterlin who is identified as the first modern economist to explore the study of economic of happiness [6]. Economics studies have grown to include many determinants such as income, occupation (unemployment, part-time, full-time, self-employed), wealth, leisure, level of skill (low, semi, highly), income inequality (low, mid, high), inflation, union membership, household size, personal (age, gender, personality, ethnicity, race), social, education, health (self-reported, health promoting behaviour, objective health status), religion, relationships (married, single, widowed, divorced, separated), social capital (social networks, norms and trust), life perceptions (financial satisfaction), having children, social relationships (family and friends), crime, area of residence (urban or rural), and many other determinants [11, 19, 20, 23].

The most common determinants are the following: inequality, age, gender, race, wealth, absolute or relative income, education, employment, number of children, place of residence, marital status, social capital, religion, and health. The aim of this economic literature is to find out how these determinants/correlates^1^ associate with happiness/life satisfaction/subjective well-being.

These determinants/correlates may associate with subjective well-being differently depending on population or data used. But many studies tend to find these common effects – an increase in wealth, education (especially at higher levels of education), income (absolute income in low-income level households and relative income in high income level households), social capital, being married, employed, living in an urban area, and being healthy tend to be associated with an increase subjective well-being [21, 24–33]. Whereas socio-economic elements like unemployment and inequality which increase the pain element of hedonic tend to have a negative relationship with SWB.

Age has a u-shaped relation with SWB meaning younger and older population tend to report higher level of life satisfaction than middle aged population [24, 26, 28, 30, 31, 33]. There is no uniform or common relationship between SWB and these determinants: race and gender. For instance, males are happier in one study whilst females are happier in another study [24–27, 30, 31, 33]. Whites tend to be happier in South Africa due to racially motivated inequalities [26].

Literature may use subjective or objective measures to quantify health as a dependent variable. Subjective measures involve self-reported heath statuses whereas objective measures include Body Mass Index and diagnosed illnesses [8, 11, 34]. Subjective measures are viewed as an effective way of measuring health [5, 8, 10, 11, 35] and are commonly used [14]. Subjective and objective measures of health are also found to be highly correlated by previous studies [36]. Hence, the study will opt for subjective health measures.

Health is also studied as an outcome variable that is dependent on certain determinants including SWB [10, 11, 37, 38]. Determinants of SWB and health are relatively similar and tend to behave differently or similarly across these variables [36]. Inequality, age, being female tend to be associated with a decrease in health whereas education, income, employment and SWB tend to be associated with an increase in health [5, 10, 11, 34, 37–39].

The prime interest of this paper is the relationship between SWB and health. Health is seen as one of the most vital determinants/correlates of SWB [6, 7]. Hence, there has been many studies investigating the relationship of these variables [8, 10, 11, 36–38, 40]. Health as an independent variable can also be measured in subjective or objective terms. Researchers tend to favour subjective health measures citing that it is a better predictor of happiness than objective measures [8, 21]. These studies [8, 21] found that subjective measures tend to be better predictors of SWB and objective measures are sometimes weakly correlated to SWB [8].

Many studies show a positive relationship between health (physical and psychological) and subjective well-being, where psychological health is highly correlated to subjective well-being than physical health [6, 8–11, 13, 14, 20, 37, 38]. Looking at the hedonic view of subjective well-being, health influence subjective well-being through the seeking of pleasure and avoiding of pain components. Illness tends to come with pain and is an interruption to one’s life [9], whereas being healthy may be associated with pleasure, positively influencing subjective well-being. These factors are found to be the influencing factors of health on subjective well-being [9].

However, many studies also found effects of subjective well-being on health in terms of mortality, longevity, and self-reported health statuses [3, 8, 9, 11]. This means that an increase in subjective well-being is associated with a decrease in the mortality rate, whereas an increase in subjective well-being is associated with longevity and positive reported health statuses. The positive effects of SWB on health are usually driven by happiness preventing autonomous nervous system from triggering physiological reactions that can have long term adverse health effects and the belief that happy people usually partake in health advancing activities [3, 7, 9, 10]. In fact, there is a positive relationship between SWB and health-promoting behaviour, where an increase in life satisfaction is associated with an increase in health-promoting behaviour [22]. This “pathway through which SWB affects health” is also found by many studies [9].

There is a bidirectional relationship between health and subjective well-being [3, 6, 8, 9], making it hard to infer causality. As much as an improvement in health is associated with an increase in subjective well-being, an increase in subjective well-being is also associated with better health. Happier people tend to be healthier (increase in subjective well-being is associated with an increase in health) or healthier people tend to be happier (increase in health is associated with an increase in happiness/subjective well-being/life satisfaction). However, some studies [40] do not find evidence of bidirectionality amongst older adults.

There is vast literature which explores the relationship between SWB and perceived health. There is also literature which takes into cognisance the moderating effects of population characteristics. However, most of this literature is based on sampling the national population. Population characteristics, access to health services and health outcomes may differ regionally with the Eastern Cape being amongst the provinces with poor health outcomes [41, 42]. Using the national population may not reflect such differences. Therefore, moderating effects of population characteristics which may be different regionally may remain hidden. Thus, the paper aims to investigate if the association between perceived health and SWB is moderated by population characteristics at a provincial level. The paper contributes to literature by studying the relationship between SWB and Health in the Eastern Cape, by stratifying between age; gender; rural; urban [10, 11] and different relative income levels. Initially we also wanted to stratify by former Transkei, and Ciskei since lifestyles of rural and urban concentrations differ. We were unable to stratify according to these categories since the National Income Dynamics (NIDs) data does not have local municipality or city/town data. Stratification is motivated by the factor that age, gender, and other characteristics tend to be associated with SWB or health differently, therefore “[serving] as moderators of the connection between SWB and health outcomes” [9]. Health tends to deteriorate with age [14] so it was of interest to us to investigate if health effects on SWB varies across age groups. Gender disparities exist in SWB and in health [14]; rural and urban areas differ in terms of pollution, stress levels and lifestyles [11], health expectations differ across different incomes [6]. It would also be of interest to see if health effects on SWB differ across these cited dynamics.

### Research methods

Our data source is from the National Income Dynamics Study (NIDs), South Africa’s first panel study. The intention of this data source is to track and understand gnawing effects of South Africa’s stubborn poverty by collecting data on socio-economic indicators including subjective wellbeing and health. NIDs is a survey of individuals that started with wave one in 2008 and has since collected data in five waves – wave two 2010/11, wave three 2012, wave four 2014/15 and wave five 2017. In each wave data on households, child and adults is collected using household, adult, and child questionnaires. These questionnaires are administered by fieldworkers on the same individual every cycle of data collection. We identified NIDs as an ideal source since it contained our variables of interest: subjective wellbeing and health and their most common determinants as indicated by literature – inequality, age, gender, race, wealth, absolute or relative income, education, employment, number of children, place of residence, marital status, social capital and religion.

Different datasets are generated from data collection. We were able to get access and download these datasets from the NIDs website. We only sampled from the adult, household, individual and household derived datasets since our focus was on adults. We also controlled for age by dropping all observations of persons under the age of eighteen. We proceeded and created age categories: those between the age of 18 & 19 were categorised as teenagers, 20 to 35 youth reflective of South Africa’s categorisation, 36 to 59 adults and 60+ pensioners. We used only the Eastern Cape sample by keeping Eastern Cape observations.

These datasets have the same individual identifiable by a unique identifier referred to as a *personal identifier *(*pid*)across waves. We went through the household and adult questionnaires and data in each wave to identify our variables and determinants. We merged datasets in each wave using the *pid*, thereafter we appended all waves into one dataset. We selected the following variables for our model(s) – self-report health status, life satisfaction, marital status, relative income, importance of religion, place of residence, number of children, absolute income, gender, race, education, age, and employment status. As cited above SWB and Health determinants are similar, therefore, we also followed previous studies’ [10, 11] logic and we selected and included control variables that relate to SWB and health.

Since NIDs data is collected on the same individual over a period we opted for a data panel model [24, 26, 28, 30]

$${SWB}_{it}={\beta }_{1}{SH}_{1it}+{\beta }_{2}{MS}_{2it}+{\beta }_{3} {}^{Y_{3it}}\! \left/ {}_{Y}\right.+{\beta }_{4}{RI}_{4it}+{\beta }_{5}{GT}_{5it}+{\beta }_{6}{C}_{6it}+ {\beta }_{7}\text{log}{Y}_{7it}+{\beta }_{8}{G}_{8it}+{\beta }_{9}{R}_{9it}+{\beta }_{10}{E}_{10it}+{\beta }_{11}{A}_{11it}+{\beta }_{12}{A}_{12it}^{2}+{\beta }_{13}{ES}_{13it}+{\varepsilon }_{it}$$

Where:

*SWB* is subjective wellbeing measured by life satisfaction question “Using a scale of 1 to where 1 means “*Very dissatisfied*” and 10 means “*Very satisfied*”, how do you feel about your life as a whole right now?” We opted for this question out of the two SWB questions since it asks about SWB presently. The other happiness question asks the individual to compare his/her current happiness with happiness from ten years ago.

*SH *measures health. We opted for subjective health since literature tends to favour self-reported health statuses naming it as a better predictor of SWB than objective measures of health. Individuals were asked to rate their level of health by responding to the following question “How would you describe your health at present? Would you say it is excellent, very good, good, fair, or poor?” These options were categorised as follows – 1 *excellent, *2 *very good, *3 *good, *4 *fair, *and 5 *poor. *We recoded the options to 1 *poor, *2 *fair, *3 *good, *4 *very good *and 5 *excellent, *so that an increase in *SH *is reflected by an increase in *SWB.*

*Covariates includes MS: marital status,*${}^{{Y}_{3i}}\!\left/ \!{}_{Y}\right.$: relative income, *RI*: Importance of religion, *GT:* place of residence (those who reside in communal/traditional lands are deemed as rural others deemed as urban), *C*: number of children,$\mathit{Log}Y$*:* absolute income. *G*: gender, *R:* Ethnic groups, *E:* education categories (no schooling, primary (grade R to 7/standard 5), secondary incomplete (grade 8/standard 6/form 1 to diploma with less than grade 12/Standard 10), matric (standard 10/grade 12/form 5), post-secondary (certificate with grade 12/standard. 10 to higher degree (masters, doctorate)), *A:* age categories (teenager (18 to 19 years), youth (20 to 35 years), adults (36 to 59 years) and pensioners (60), ${A}^{2}$: age squared, and *ES* employment status (not economically active, unemployed discouraged, unemployed strict and employed)*.* $\varepsilon$ – error term, Subscript $i$ and $t$ represents an individual and time respectively, and $\beta$ – coefficients.

We estimated two panel models one having SWB as a dependent variable and another SH as a dependent variable. We did this to compare determinants’ behaviour across these two variables [36] and see if there is bidirectionality. We ran a pooled OLS and a panel regression for the SWB and health status models. We tested for panel effects on both models using the Breusch-Pagan Lagrange multiplier (LM test). The SWB model was insignificant we could not reject the null of no panel effects. However, the health status model had panel effects. We therefore used the robust random effect panel estimation technique for the SWB models.

We also observed that these variables significantly determine each other.^2^ We tested for correlation using Pearson’s *r*, Pearson’s *chi*, Spearman’s, and Cramer’s *V* tests all indicating weak association. However, we still assumed bidirectionality between these two variables. We therefore saw it fit to select an instrument variable related to *SH,* but not to the error term of *SWB* and ran least squares regressions to control for potential endogeneity [8–11, 20, 37, 38]. *SH* was insignificant. We did an endogeneity test and *SH* was not endogenous. We tested the strength of our instrument variables results also indicated weak instruments.

Since we decided on a panel model, we further tested for an appropriate panel estimation technique between random and fixed effects using the Hausman test. The test revealed that the random effect estimation technique is appropriate. We therefore stuck with the random effects model and focussed on stratifying according to age categories, gender, place of residence and relative income.

## Results

### Sample summary statistics

Descriptive statistics are displayed in Table 1. Majority of the respondents reported a life satisfaction level of five and a good health status. Most people in our sample (43%) never married, are below average income (56%), female (67%), African (89%), of working age (86%) and are not employed (65%), regard religion important (91%), do not reside in the urban area (52%), have incomplete secondary education (44%).^3^ Based on the displayed data we can make some inferences which can be verified by future research. There is a clear display of inequality since most in our sample reported below average income whereas a few are above average income. Inequality is also visible in household income where average income of R4960 is far below the highest level of household income reported (R1 015 900). Majority of the participants regard religion as important testament to South Africa and Eastern Cape being a religious country and province. The Eastern Cape is predominantly rural with a young, female dominated and African population hence majority in the sample are from rural areas, young, female, and African [43]. Reported number of children are lower than provincial fertility rates which may reflect underreporting in our sample [43].

**Table 1:** Descriptive statistics

Variable	Obs	Mean	Std. Dev	Min	Max
**Life satisfaction**	5192	4.692	2.213	1	10
**Health Status**	5192	3.585	1.119	1	5
**Marital status**	.	.	.	.	.
Married	5188	.354	.478	0	1
Living with partner	5188	.039	.194	0	1
Widow/Widower	5188	.14	.347	0	1
Divorced or separated	5188	.037	.188	0	1
Never Married	5188	.43	.495	0	1
**Relative income**	.	.	.	.	.
Below average income	4989	.562	.496	0	1
Average income	4989	.366	.482	0	1
Above average income	4989	.072	.258	0	1
**Importance of religion**	.	.	.	.	.
Not important at all	5140	.026	.16	0	1
Unimportant	5140	.059	.236	0	1
Important	5140	.504	.5	0	1
Very important	5140	.411	.492	0	1
**Place of residence**	.	.	.	.	.
Rural^a^	5188	.522	.5	0	1
Urban	5188	.478	.5	0	1
Number of children	5192	1.769	2.026	0	12
Household income	5192	4960.701	15,623.428	71.112	1,015,900
**Gender**	.	.	.	.	.
Male	5192	.332	.471	0	1
Female	5192	.668	.471	0	1
**Ethnicity**	.	.	.	.	.
African	5192	.893	.309	0	1
Coloured	5192	.09	.286	0	1
White	5192	.017	.13	0	1
**Education categories**	.	.	.	.	.
no schooling	5181	.094	.292	0	1
primary	5181	.274	.446	0	1
secondary incomplete	5181	.439	.496	0	1
Matric	5181	.117	.322	0	1
post-secondary^b^	5181	.075	.263	0	1
**Age categories**	.	.	.	.	.
teenager	5191	.037	.189	0	1
youth	5191	.33	.47	0	1
adult	5191	.448	.497	0	1
pensioner	5191	.185	.388	0	1
**Employment status**	.	.	.	.	.
Not Economically Active	5180	.486	.5	0	1
Unemployed Discouraged	5180	.03	.17	0	1
Unemployed Strict	5180	.137	.344	0	1
Employed	5180	.347	.476	0	1

^a^Communal land dwellers

^b^Matric plus certificate to PhD

### SWB and Health random effects regressions

We estimated random effects regressions for SWB and health status, results are shown in Table 2. Health status has a positive relationship with SWB (Table 2, column 1). The results also indicate a positive effect of subjective well-being (SWB) on health status, suggesting that a one-unit increase in life satisfaction is associated with the probability of having better health status by 2.95% (column 2).

**Table 2:** SWB and health random effects regression

	(1)	(2)
VARIABLES	SWB	Health status
Fair	0.570***	
	(0.160)	
Good	0.550***	
	(0.149)	
Very Good	0.803***	
	(0.156)	
Excellent	0.752***	
	(0.162)	
Living with partner	-0.148	0.120
	(0.157)	(0.0778)
Widow/Widower	-0.0322	0.0341
	(0.0932)	(0.0520)
Divorced or separated	-0.264*	0.0356
	(0.151)	(0.0828)
Never Married	-0.252***	0.0312
	(0.0752)	(0.0426)
Average income	1.013***	0.0458
	(0.0625)	(0.0320)
Above average income	0.439***	0.229***
	(0.131)	(0.0567)
Unimportant	0.141	-0.308***
	(0.224)	(0.102)
Important	0.198	-0.241***
	(0.204)	(0.0886)
Very important	0.756***	-0.0564
	(0.208)	(0.0899)
Urban	0.0877	-0.136***
	(0.0644)	(0.0360)
Number of children	-0.0122	0.0166
	(0.0199)	(0.0112)
Household income	0.238***	0.0103
	(0.0366)	(0.0179)
Female	-0.0343	-0.191***
	(0.0804)	(0.0449)
Coloured	0.637***	-0.0800
	(0.118)	(0.0602)
White	1.435***	0.153
	(0.263)	(0.127)
Primary	-0.147	0.0297
	(0.116)	(0.0614)
Secondary	-0.0786	0.231***
	(0.121)	(0.0641)
Matric	0.0947	0.355***
	(0.147)	(0.0780)
Post secondary	0.164	0.350***
	(0.166)	(0.0867)
Age	-0.0211**	-0.0262***
	(0.0103)	(0.00561)
Age squared	0.000248**	3.14e-06
	(0.000105)	(5.72e-05)
Unemployed Discouraged	0.295*	0.0574
	(0.162)	(0.0833)
Unemployed Strict	0.428***	0.0844*
	(0.0913)	(0.0449)
Employed	0.317***	0.175***
	(0.0758)	(0.0369)
Life satisfaction		0.0295***
		(0.00691)
Constant	1.632***	4.525***
	(0.455)	(0.214)
Observations	4,912	4,912
Number of pid	1,423	1,423

Standard errors in parentheses *** *p* < 0.01, ** *p* < 0.05, * *p* < 0.1

The probability of having high level of life satisfaction for divorcees and never married individuals is reduced by 26% and 25% respectively. Age is u-shaped showing a non-linear relationship with SWB. Absolute and relative income are positively associated to SWB reflective of the characteristic of the population – below average income. Interestingly, average relative income significantly increases life satisfaction by more than 100%, while having a high income significantly increase life satisfaction by 44%. In addition, those who think religion is very important are more likely to have an increase in life satisfaction (76%). Relative to Africans, the probability having increased life satisfaction for Coloured individuals and Whites is increased by 63% and more than 100% respectively compared to other ethnic groups. Employment status is also positively associated with life satisfaction, being strictly unemployed or employed increases the probability of life satisfaction.

Above average relative income is positively associated with health status. This means that the probability of being healthy is increased by 22% for those with above average income relative to those with below average income. Being an urban dweller, female, older, and having religious affiliation were also found to have a negative association with the health status, whereas being educated and employed were positively associated with it.

### SWB ordered probit regressions stratified by age and sex

Table 3 presents marginal effects on SWB of all measured covariates, including health status, disaggregated by age. There were insufficient observations for the teenager category, hence, there are no results under that classification. Health status is found to be significant at all levels of age, becomes increasingly significant at higher age categories, signifying that as health deteriorates, it becomes a significant contributor to SWB (Columns 1–3). Further illuminating our premonitions, if health varies with age, health effects on SWB varies with age.

**Table 3:** Random effect regression for SWB stratified by age

VARIABLES	(1) Youth	(2) Adult	(3) Pensioner
**Health Status**			.
Fair	1.71*** (.442)	.321 (.226)	.615*** (.233)
Good	1.219*** (.357)	.445** (.213)	.618*** (.232)
Very Good	1.61*** (.35)	.607*** (.218)	.624** (.276)
Excellent	1.534*** (.352)	.609*** (.229)	.895** (.381)
**Marital Status**			.
Living with partner	-.427 (.287)	-.059 (.222)	-.027 (.399)
Widow/Widower	-.31 (.408)	-.088 (.13)	.167 (.162)
Divorced or separated	-1.126 (.761)	-.301* (.178)	.161 (.269)
Never Married	-.335** (.142)	-.201* (.105)	-.312 (.202)
**Relative income**			.
Average income	1.068*** (.111)	.999*** (.094)	.824*** (.136)
Above average income	.702*** (.234)	.349* (.184)	-.075 (.327)
**Importance of religion **			.
Unimportant	.333 (.312)	-.057 (.353)	-.02 (.823)
Important	.178 (.271)	.194 (.329)	.011 (.769)
Very important	.676** (.279)	.852*** (.328)	.467 (.792)
**Place of residence**			.
Urban	-.005 (.102)	.018 (.096)	.445** (.175)
Number of children	-.122** (.051)	0 (.028)	.013 (.04)
Household income	.105* (.061)	.316*** (.051)	.21* (.123)
**Gender**			.
Female	.235* (.125)	.029 (.132)	-.56** (.216)
**Ethnicity**			.
Coloured	.948*** (.187)	.442** (.173)	.137 (.335)
White	2.642*** (.667)	.696** (.27)	1.802*** (.596)
**Education categories **			.
primary	-.864* (.499)	.032 (.167)	-.304* (.169)
secondary incomplete	-.887* (.48)	.106 (.167)	-.092 (.204)
Matric	-.79 (.496)	.352* (.208)	.244 (.467)
post-secondary	-.905* (.519)	.449** (.218)	.03 (.537)
**Employment status**			.
Unemployed Discouraged	.612** (.249)	.125 (.226)	.607 (.994)
Unemployed Strict	.731*** (.127)	.271* (.157)	.186 (.597)
Employed	.597*** (.128)	.266** (.107)	.121 (.232)
Constant	2.16*** (.776)	.558 (.565)	2.191* (1.235)
Mean dependent var	4.705	4.734	4.670
Overall r-squared	0.180	0.210	0.146
Chi-square	363.754	618.685	189.051
R-squared within	0.099	0.077	0.077
SD dependent var	2.127	2.218	2.106
Number of observation	1628	2204	912
Prob > chi2	0.000	0.000	0.000
R-squared between	0.256	0.385	0.173

Standard errors in parentheses *** *p*<0.01, ** *p*<0.05, * *p*<0.1

Marital status influence SWB differently across age groups. The youth and adults those who maybe at an age where marriage is important, being divorced or separated or never married is associated with lower SWB, whereas it is insignificant for pensioners. Average income is associated with positive SWB consistently across all age categories whereas, above average income is insignificant to SWB for pensioners. Religion is significantly related to SWB in all age groups besides pensioners. Being a pensioner and leaving in an urban area increases a person’s SWB maybe due to access to better services including health facilities. Having children is associated with a decrease in SWB for the Youth, since children may hinder that age group from conducting certain activities freely. Being female is negatively related to SWB for the pensioners, whereas it is positive for the youth. Being Coloured and White is significantly associated to positive SWB compared to Africans, however, being a Coloured pensioner does not seem to influence SWB. As a youth, having primary education and incomplete secondary school is negatively related to SWB, signifying the importance of progressing educationally for that age group. However, having post-secondary education is also associated with less SWB, maybe a consequence of high youth unemployment amongst graduates. Educational progression is associated with an increase in SWB for the adult group. Being employed consistently is associated with an increase in one’s SWB in all age categories besides pensioner, which may be because they are not of working age. Thus, determinants associate different with SWB at different age groups indicating the value of each determinant across different age groups.

Turning into the SWB model that is disaggregated by sex (Table 4), health status is insignificant for males, yet it is positively significant for females. Like age categories those who report lower health status seemingly their health status is positively related to SWB. Thus, gender differences in health status are also associated with different effects of health on SWB Tables 5 and 6.

**Table 4:** Random effect regression for SWB stratified by gender

VARIABLES	(1) Female	(2) Male
**Health Status**		
Fair	.673*** (.178)	.11 (.346)
Good	.624*** (.164)	.186 (.335)
Very Good	.932*** (.172)	.327 (.346)
Excellent	.838*** (.182)	.349 (.349)
**Marital Status**		
Living with partner	-.083 (.194)	-.281 (.267)
Widow/Widower	.022 (.102)	-.041 (.301)
Divorced or separated	-.185 (.176)	-.399 (.296)
Never Married	-.148 (.091)	-.473*** (.144)
**Relative income**		
Average income	.972*** (.074)	1.086*** (.115)
Above average income	.671*** (.162)	.046 (.217)
**Importance of religion**		
Unimportant	-.604 (.4)	.407 (.266)
Important	-.433 (.356)	.356 (.243)
Very important	.098 (.357)	1.017*** (.256)
**Place of residence**		
Urban	.061 (.081)	.093 (.108)
Number of children	-.009 (.022)	.
Household income	.255*** (.045)	.202*** (.064)
**Ethnicity**		
Coloured	.727*** (.147)	.41** (.195)
White	1.521*** (.273)	1.14 (.739)
**Education categories**		
primary	-.06 (.133)	-.368 (.231)
secondary incomplete	-.053 (.14)	-.189 (.237)
Matric	.003 (.177)	.165 (.269)
post-secondary	.155 (.198)	.085 (.307)
Age	-.019 (.013)	-.038** (.017)
Age Squared	0 (0)	0** (0)
**Employment status**		
Unemployed Discouraged	.287 (.193)	.39 (.282)
Unemployed Strict	.465*** (.11)	.367** (.162)
Employed	.336*** (.094)	.338*** (.129)
Constant	1.882*** (.619)	2.774*** (.76)
Mean dependent var	4.702	4.724
Overall r-squared	0.180	0.179
Chi-square	736.275	.
R-squared within	0.081	0.081
SD dependent var	2.150	2.191
Number of observation	3291	1621
Prob > chi2	0.000	.
R-squared between	0.333	0.302

Standard errors in parentheses *** *p* < 0.01, ** *p* < 0.05, * *p* < 0.1

**Table 5:** Random effect regression for SWB stratified by place of residence

VARIABLES	(1) Rural	(2) Urban
**Health Status**		
Fair	.659*** (.185)	.422 (.258)
Good	.657*** (.182)	.381 (.234)
Very Good	1.033*** (.187)	.51** (.245)
Excellent	.796*** (.195)	.649** (.255)
**Marital Status**		
Living with partner	.484* (.284)	-.323* (.183)
Widow/Widower	-.105 (.106)	.119 (.182)
Divorced or separated	-.038 (.214)	-.427** (.215)
Never Married	-.161 (.101)	-.339*** (.113)
**Relative income**		
Average income	.9*** (.079)	1.124*** (.1)
Above average income	.395** (.201)	.478*** (.173)
**Importance of religion**		
Unimportant	.3 (.26)	-.037 (.42)
Important	.378 (.239)	.006 (.384)
Very important	.849*** (.243)	.634 (.389)
Number of children	-.013 (.024)	-.004 (.04)
Household income	.151*** (.051)	.287*** (.053)
**Gender**		
Female	-.038 (.109)	-.004 (.125)
**Ethnicity**		
Coloured	.945*** (.094)	.569*** (.121)
White	0 (0)	1.252*** (.258)
**Education categories**		
primary	-.247* (.129)	.151 (.23)
secondary incomplete	-.131 (.14)	.157 (.229)
Matric	-.028 (.193)	.364 (.248)
post-secondary	.3 (.24)	.287 (.265)
Age	-.009 (.012)	-.054*** (.02)
Age Squared	0 (0)	.001*** (0)
**Employment status**		
Unemployed Discouraged	.333 (.208)	.278 (.258)
Unemployed Strict	.57*** (.114)	.357** (.148)
Employed	.216** (.101)	.447*** (.116)
Constant	1.827*** (.596)	1.986*** (.757)
Mean dependent var	4.477	4.961
Overall r-squared	0.116	0.214
Chi-square		671.346
R-squared within	0.076	0.090
SD dependent var	1.940	2.357
Number of observation	2555	2357
Prob > chi2		0.000
R-squared between	0.181	0.378

Standard errors in parentheses *** *p* < 0.01, ** *p* < 0.05, * *p* < 0.1

**Table 6:** Random effect regression for SWB stratified by relative income

VARIABLES	(1) Above average	(2) Average income	(3) Below average income
**Health Status**			
Fair	.796 (.602)	.682** (.315)	.519*** (.196)
Good	.439 (.552)	.874*** (.302)	.423** (.177)
Very Good	1.029* (.554)	.884*** (.315)	.813*** (.187)
Excellent	.483 (.55)	.801** (.325)	.869*** (.197)
**Marital Status**			
Living with partner	.415 (.745)	-.727** (.3)	.009 (.195)
Widow/Widower	.085 (.397)	-.168 (.156)	.057 (.122)
Divorced or separated	.097 (.571)	-.281 (.244)	-.252 (.21)
Never Married	-.838*** (.297)	-.357*** (.123)	-.121 (.103)
**Importance of religion**			
Unimportant	-.298 (.918)	-.684* (.402)	.331 (.265)
Important	-.286 (.808)	-.981*** (.366)	.576** (.235)
Very important	.702 (.822)	-.329 (.37)	.963*** (.239)
**Place of residence**			
Urban	-.201 (.263)	.461*** (.106)	-.077 (.085)
Number of children	-.074 (.09)	.01 (.035)	-.029 (.024)
Household income	.19 (.149)	.094 (.058)	.308*** (.048)
**Gender**			
Female	.61* (.329)	-.178 (.128)	-.009 (.107)
**Ethnicity**			
Coloured	1.224*** (.427)	.286* (.159)	.75*** (.197)
White	1.756*** (.484)	1.058*** (.304)	2.119 (1.387)
**Education categories**			
primary	.724 (.502)	-.252 (.197)	-.114 (.141)
secondary incomplete	.338 (.478)	-.044 (.211)	-.055 (.147)
Matric	1.346** (.564)	.218 (.244)	-.069 (.191)
post-secondary	1.713*** (.584)	.127 (.25)	-.232 (.276)
Age	-.125** (.054)	-.036** (.016)	.002 (.013)
Age Squared	.001* (.001)	0** (0)	0 (0)
**Employment status**			
Unemployed Discouraged	.882 (.986)	.809*** (.233)	.014 (.217x)
Unemployed Strict	.561 (.497)	.459*** (.163)	.396*** (.111)
Employed	.358 (.319)	.386*** (.117)	.294*** (.104)
Constant	4.705** (1.959)	5.08*** (.78)	.281 (.564)
Mean dependent var	5.079	5.513	4.134
Overall r-squared	0.340	0.119	0.073
Chi-square	251.230	239.323	210.004
R-squared within	0.213	0.036	0.055
SD dependent var	2.431	1.997	2.051
Number of obs	354	1806	2752
Prob > chi2	0.000	0.000	0.000
R-squared between	0.339	0.162	0.118

Standard errors in parentheses *** *p* < 0.01, ** *p* < 0.05, * *p* < 0.1

Marital status is insignificant for females but being never married and male is associated with a decrease in SWB. In some rural Eastern Cape societies being an unmarried man comes with its own challenges which associate with less SWB. However, this may also be true for females. Relative income, absolute income, strictly unemployed and employed are positively associated with SWB for both males and females. This positive relationship between unemployment and SWB is still a puzzle since unemployment is negatively related to SWB. Surprisingly, religion positively associates with SWB for males and not for females whom one would assume are more religious than men. Age is u-shaped for males, being Coloured is positively associated with SWB even when stratified.

### SWB regressions stratified by place of residence and income level

Based on our sampled results, rural residents have lower SWB, and health status compared to urban residents. Health status is positive and significantly related to SWB at all levels of health statuses in the rural area. It is positively associated with SWB at higher levels of health in the urban area. This may be reflective of health status disparities that exist amongst place of residences.

Living with a partner is positive and significantly related to SWB for rural residence, whilst being divorced or separated and never married are negative and significantly related to SWB for an urban dweller. Relative income, absolute income, strictly unemployed, employed, is positive and significantly related to SWB in rural and urban areas. Being a non-African in an urban area is positively related to SWB. This is because in our sample there are no White people in rural areas. Having primary education and residing in a rural area is negatively associated with SWB.

Those who reported lowest income relative to others have the lowest SWB and health status. Health status influence SWB differently at different income classifications. Further illuminating the fact that different population characteristics act as moderators influencing how health associates with SWB. Individuals with below average and average income positively associates with SWB at all levels of health. Reemphasising that those who report low levels of health like below average income participants find solace in positive health outcomes. Those who have below average income the absolute income is positively associated with SWB as expected. Poor people generally find religion important, hence, we also find that Importance of religion is positively associated with SWB for those who reported below average income. There is a higher probability of having higher SWB amongst Coloureds compared to other ethnic groups across all income levels. In addition, both employment and unemployment positively influence SWB for individuals reporting below average and average income levels.

## Discussion

Even though our literature review is totally subjective some of our findings are like the previous empirical evidence. We estimated robust random effects regressions for SWB and health status. Additional lifestyle variables were controlled in the health status model and the rest of the selected variables were the same for both models [8, 10, 11, 36]. Health status has a positive relationship with SWB at all levels of positive health status confirming previous studies’ findings [8, 21, 24–26, 28, 29, 31, 33]. It is also highly significant confirming that health is an important determinant of SWB [6, 7]. The positive relationship displayed in our regression between SWB and health in the hedonic view may be due to decreased pain and increased pleasure as a health increase is associated with higher SWB [9].

Looking at the 2nd column of Table 2 which is the health status regression, SWB also has a positive relationship confirming findings from to other studies [3, 8–11, 37, 38]. As reflected in our literature review, the positive effects of SWB on health may be due to positive physiological reactions affecting health positively [3, 7, 9, 10].

What we can gather from these finding is that health influences SWB and SWB also determines health. We can therefore presume that there is some correlation between the two variables. We ran tests of correlation which confirmed existence of a relationship between these two variables, however, the test showed a weak correlation in our study. Majority of those with poor health reported low levels of life satisfaction compared those with a better health status who reported an average (level five) life satisfaction. This further confirms that those who are less healthy usually report less SWB.

The following determinants are positively related to SWB: married, relative income, religion, absolute income, non-African, confirming previous findings [8, 24, 26–31, 36, 37]. Majority of our sample is not from higher income households which is why both absolute and relative income influence SWB. Age is u-shaped showing a non-linear relationship between age and SWB [24, 26, 28, 30, 31]. Both those who are unemployed and employed show a positive relationship with SWB of which unemployment should not be associated with higher SWB. Majority of the sample is unemployed, meaning there is high unemployment, therefore, the negative effects of unemployment on SWB maybe diminished [35]. In a society like the Eastern Cape where unemployment is high, people tend to be accustomed to their situation, thus, they return to their baseline level of SWB and the stigma that may adversely affect SWB is low since most people are unemployed [35].

SWB and health status have similar determinants of which some of these determinants may be associated with both higher SWB and health. We found the following determinants to be associated with both higher SWB and health: relative income and employment similar to previous studies [36]. We did not find a negative relationship between health and unemployment confirming previous longitudinal data studies [35]. Having higher income, being employed is associated with higher health and subjective wellbeing as it affords one better medical care. Higher SWB and health ought to be associated with higher levels of education [5, 10, 37], but we only found education to be significant with health. Education may afford a person a higher income, better one’s chances of being employed and may be associated with a better understanding of the health system [37], therefore increasing one’s health status.

Being married does not influence a person’s health status as shown in Table 2 and that is the general trend in literature. We also found being female to be associated with a less health status like previous studies [11, 36, 37]. However, it is important to note that men generally overstate their health statuses in fear of being ridiculed by society. This is not far off since the Eastern Cape is a patriarchal society where men should not be seen as weak or frail. Age is negatively related to health since a person’s health deteriorates with age [10, 11, 14, 36, 37]. Living in an urban area is negatively related to health and an increase in children increases one’s health status. Most of the literature reviewed in this paper did not find any significant relation between health and geographical type and number of children.

When we stratified our data into different characteristics (age, sex, place of residence and income levels) we found that health effects on SWB varied with all different characteristics. Effects of health on SWB differed according to gender [44]. Determinants of SWB including health associated with SWB differently amongst different races in South Africa [26]. Although, literature used different characteristics to ours, our findings further confirm that population characteristics act as moderators of determinants’ effects including health’s on SWB.

We faced some limitations, one being that NIDs is not recommended for provincial studies. We could not stratify for Transkei and Ciskei since our data source did not collect town or local municipality data. We also could not prove that health is an endogenous variable. Therefore, failing to control for any possible endogeneity that may arise due to the bidirectional relationship between SWB and health. The health status assessed in this study relies on self-reported data. Literature suggests that self-reported data may be subject to inaccuracies, as some respondents might be reluctant to disclose their true health status due to stigma, while others may exaggerate it to justify economically active decisions.

## Conclusion

Subjective wellbeing and health are related. Indeed, to achieve full health one must achieve SWB. When considering health improvement policies, one must consider SWB to realise health as dictated by WHO and UN SDG 3.

There are determinants that increase both SWB and health. These are income and employment. Thus, to improve SWB and health which is equivalent to how WHO and UN view health, policies need to improve income and employment outcomes. These findings may also inform economic outcomes related to labour markets, economic growth, and development.

This was our first attempt in providing provincial or sub-regional SWB studies even though NIDs is not conducive for provincial studies. Our findings confirm national studies’ findings. Provinces differ in terms of health services and outcomes. National studies of SWB and health may not reflect these differences. This was our valiant effort of revealing this problem. Thus, SWB and health improvement policies need to be cognisant of such national and provincial disparities. Health and other determinants of SWB were found to be associated with SWB differently when different population characteristics were considered. Population characteristics moderated how health associates with SWB. Well-being improvement policies need to be cognisant of provincial and population characteristics differences.

## Data Availability

The data analysed in this study is available from the SALDRU https://www.nids.uct.ac.za/.

## Abbreviations

SWB: Subjective wellbeing
SH: Health status
NIDS: National Income Dynamics Study
WHO: World Health organisation
UN: United nations

## Footnotes

1 Determinant are based on causation while correlates are based on correlation studies [14].

2 Results are displayed in the results and discussion section.

3 Consistent with mean years of education as per https://globaldatalab.org/areadata/profiles/ZAFr102/.
